# To Group or Not to Group? Good Practice for Housing Male Laboratory Mice

**DOI:** 10.3390/ani7120088

**Published:** 2017-11-24

**Authors:** Sarah Kappel, Penny Hawkins, Michael T. Mendl

**Affiliations:** 1 Bristol Veterinary School, Bristol University, Langford House, Langford BS40 5DU, UK; Mike.Mendl@bristol.ac.uk; 2 Research Animals Department, RSPCA, Wilberforce Way, Southwater, West Sussex RH13 9RS, UK

**Keywords:** refinement, mouse welfare, mouse husbandry, mouse aggression, male mice, social organisation, group housing, single housing, animal husbandry, animal welfare, animal management

## Abstract

**Simple Summary:**

Wild mice live in territories inhabited by one adult male, several females, and their offspring. This cannot be replicated in the laboratory, so male mice are usually housed in single-sex groups or individually. However, there can be serious animal welfare problems associated with both these approaches, such as lack of social contact when housed individually or aggression between males when kept in groups. Group housing is widely recommended to give male laboratory mice the opportunity to behave as ‘social animals’, but social stress can be detrimental to the welfare of these animals, even without injurious fighting. All of this can also affect the quality of the science, giving rise to ethical concerns. This review discusses whether it is in the best welfare interests of male mice to be housed in groups, or alone. We conclude that it is not possible to give general recommendations for good practice for housing male laboratory mice, as responses to single- and group-housing can be highly context-dependent. The welfare implications of housing protocols should be researched and considered in each case.

**Abstract:**

It is widely recommended to group-house male laboratory mice because they are ‘social animals’, but male mice do not naturally share territories and aggression can be a serious welfare problem. Even without aggression, not all animals within a group will be in a state of positive welfare. Rather, many male mice may be negatively affected by the stress of repeated social defeat and subordination, raising concerns about welfare and also research validity. However, individual housing may not be an appropriate solution, given the welfare implications associated with no social contact. An essential question is whether it is in the best welfare interests of male mice to be group- or singly housed. This review explores the likely impacts—positive and negative—of both housing conditions, presents results of a survey of current practice and awareness of mouse behavior, and includes recommendations for good practice and future research. We conclude that whether group- or single-housing is better (or less worse) in any situation is highly context-dependent according to several factors including strain, age, social position, life experiences, and housing and husbandry protocols. It is important to recognise this and evaluate what is preferable from animal welfare and ethical perspectives in each case.

## 1. Introduction

It is increasingly accepted that, if animals are able to achieve their wants and needs, they will be less stressed, with better welfare, which will lead to more valid, translatable science [1,2,3,4,5]. This is reflected in legislation, e.g., the European Union (EU) Directive regulating the care and use of animals in research and testing requires Member States to ensure that ‘*any restrictions on the extent to which an animal can satisfy its physiological and ethological needs are kept to a minimum*’ [6]. Some needs are well-evaluated and simple to address, such as the provision of adequate nesting material [7,8], but others are less straightforward—and we believe that the social ‘needs’ of male mice fall into the latter category.

In this paper, we describe the natural social behaviour of the male mouse, explain why this cannot feasibly be replicated in the laboratory, and discuss the pros and cons of different housing protocols, broadly divided into ‘individual’ and ‘group’ housing. The fundamental issue we consider is whether it is in the best welfare interests of male mice to be housed together, or alone. Aggression is a primary consideration when housing mice in general [9] and injurious aggression, or the lack of this, is frequently used as the primary indicator of success when group-housing male mice [9,10,11,12]. However, the absence of aggression does not, in itself, mean that all the animals within a group are in a state of positive welfare. This review discusses the likely impacts, positive and negative, of single and group housing, identifying potential welfare indicators to enable better-informed decision-making regarding housing protocols.

We also include some results from a survey of people directly involved in housing, caring for, and using male mice in the laboratory, as we aimed to explore current practice, awareness of mouse behaviour, and aspirations for mouse housing. Finally, we propose some action points for good practice on the basis of current knowledge and pose some research questions, all for consideration by scientists, animal technologists, regulators, animal care and use committees (such as the UK Animal Welfare and Ethical Review Body, or AWERB), and funding bodies.

### The Survey of Current Practice

Briefly, a survey was designed using Google forms, which ran throughout April 2017, and was circulated via social media, colleagues in the field, and online discussion forums. It was aimed at a range of stakeholders including scientists, animal technologists, veterinarians, and members of AWERBs, with the objective of ascertaining current practice, welfare issues, and views regarding housing male mice. There were 147 responses, mainly from the UK, with most respondents identifying themselves as animal technologists (79 people), Named Animal Care and Welfare Officers (43), AWERB members (39), or scientists (29) (more than one response was permitted to this question). Most worked in universities (82 respondents) with large numbers of animals; almost 30% of respondents worked in facilities with over 10,000 mice. The survey, with more detail regarding the responses, is set out in the Supplementary Material File, and some relevant results are included within the rest of this paper.

## 2. Natural Mouse Behaviour

Free-living mice from which laboratory strains were derived (*Mus musculus*) form territories, with each territory inhabited by a deme (or small population) comprising a dominant male, several females, and their pups and non-dispersing juveniles [13]. Territory size depends on food availability and population density, with home ranges varying from a few square metres for commensal demes inhabiting areas around human dwellings (e.g., farms, buildings, food stores; [14]), up to several square kilometres for colonies in natural habitats and not living commensally with humans [15,16]. Sexually mature males either disperse or stay to inherit the parental territory, depending on population density and the size and aggressiveness of each young male [13]. Smaller males usually disperse and often become non-territory holders [17]. Resident males are highly intolerant of intruders, or other dominant males who try to compete for territory, and the presence of a stranger provokes persistent chasing and aggressive behaviour in the territory holder [18,19,20]. Communication through scent cues deposited in the environment is particularly important in maintaining social systems, enabling animals to recognise individuals, their social status, and the territory they inhabit [21]. 

Although laboratory mice have been bred (and usually inbred) in captivity for many generations, there is scientific evidence that the above behaviours may still be innate. Studies in domesticated species have demonstrated that wild-type behaviours continue to be expressed under naturalistic conditions, e.g., nest building behaviour in sows [22] and exploration behaviour in laboratory rats [23]. In the latter example, laboratory rats released into a semi-wild environment rapidly expressed many wild-type behaviours, and there is convincing evidence that domestication has also left the natural behaviour of mice largely unchanged [16,24]. This is likely to have implications for the ability of laboratory mice to satisfy their physiological and ethological needs in ‘standard’ laboratory housing, the design of which is strongly influenced by human requirements for standardisation, ease of cleaning, and manageable economic costs [3].

On this issue, the survey asked whether respondents believed that ‘male mice naturally prefer to live with other mice’. From a total of 147 respondents, 120 answered ‘yes’, 20 answered ‘no’, and 7 did not know. This suggested a reasonable level of awareness of natural mouse behaviour amongst respondents, with 72 of 147 selecting the correct response that male mice in the wild live with a group of female mice and their offspring, while 58 believed that they lived with a group of other males and females, with their offspring. 

## 3. Codes of Practice for Mouse Housing, Husbandry and Care

Recognising that wild mice have a complex social organisation in which a territory is inhabited by one adult male, several females, and their offspring, most Codes of Practice classify *Mus musculus* as a ‘social species’ and recommend that laboratory mice are kept in stable groups, regardless of gender. For example, the UK Home Office Code of Practice advises that, for all rodents, ‘*gregarious species should be group*-*housed as long as the groups are stable and harmonious—social housing is vitally important to the welfare of social species and strains*’. It also mentions that it may be difficult to achieve harmonious groups of males of some strains of mice due to the risk of aggression, suggests that expert advice is sought in order to group these successfully, and permits single housing if adverse effects or injuries are likely ([25] Section 3, Chapter 2, para 1.3.1; see also [26]). The US Guide states that ‘*single housing of social species should be the exception*’ but does note that ‘*in some species, social incompatibility may be sex biased; for example, male mice are generally more prone to aggression than female mice*’, before listing ways of reducing the risk of social incompatibility [27].

### 3.1. Do Codes of Practice Reflect ‘Natural’ Mouse Behaviour?

The default position within the above Codes of Practice that social species should be group-housed is undoubtedly both humane and ethical, with provisos relating to appropriate group formation, consistency, and surveillance, in the context of a good quality and quantity of space. However, it is unclear to the authors how the term ‘social’ ought to be defined for male mice. Presumably, the preferred housing protocol from the male mouse’s perspective would be with a harem of females, incorporating sufficient space for juvenile male offspring to be driven away—which is not feasible in research and testing laboratories for obvious reasons. As the male mouse is pre-adapted to live with other mice, does this necessarily mean that living with other males is the next best thing? 

Most survey respondents believed that male mice should, ideally, be group-housed in the laboratory (123 people, as opposed to 12 who disagreed), but there is debate about this. Some researchers have suggested that housing male mice together is not natural, and may be stressful, as male mice do not share territories in the wild [18,19,20]. Free-living male mice are also rarely subjected to the stress of social defeat, as they tend to be territorially isolated [28], whereas artificially group-housed males may be socially defeated many times every day. In contrast, others argue that aggression is a natural behaviour, whereas living alone is not [29]. Encountering these differing viewpoints prompted the authors to undertake this review.

### 3.2. Animal Welfare, Scientific and Ethical Implications

The debate on male mouse housing deserves consideration for several reasons. Although aggression is indeed a natural behaviour, in the confines of standard laboratory housing, injurious aggression between male mice can cause severe stress, pain, or even death (reviews in [9,12]). This is a fundamental concern with respect to group-housing male mice, although it is not the only welfare issue. 

The occurrence of repeated social defeat, unnatural despot or subordinate social groups, and differences in physiology and behaviour between dominant and subordinate male mice, all raise concerns about the welfare of group-housed male mice (particularly subordinates), even in the absence of fighting that causes significant injury. Whatever the apparent level of aggression, an individual’s position in the hierarchy can also have wide-reaching effects, as there is evidence that subordinate male mice differ in their physiology and behaviour from dominant males (reviewed in [28,30]), which may also negatively affect the welfare of the subordinate individuals. 

However, the welfare of individually housed male mice may be significantly diminished by the complete lack of social interactions [29]. The central question is therefore whether, all things considered, male mice are likely to have a better welfare when housed either individually or in groups. 

Housing that does not permit desirable natural behaviours, or causes stress, can also introduce confounds that will affect the quality of the science (see Section 4; [31,32]). This is a serious ethical issue, because experimental results with poor validity, reproducibility, and translatability waste animals’ lives, as well as hamper medical progress if the purpose of the study is applied medical or veterinary research [2,33,34]. There are also implications for staff who are interested in promoting a good ‘culture of care’ at the establishment, if they feel that the housing protocols do not afford animals an acceptable quality of life.

### 3.3. The Concept of ‘Quality of Life’

How do we define and assess an animal’s quality of life? Emotional (affective) states (relatively long-lasting mental ‘mood’ states that last longer than the rapid emotional responses or feelings that are induced by a stimulus) are viewed as critical determinants of animal well-being [35,36,37,38,39,40,41,42]. If emotions are considered to be states induced by ‘rewards’ and ‘punishers’ [43], then chronic or multiple exposures to a reward or punisher leads to a positively valenced (good) affective state or negatively valenced (poor) affective state respectively. These affective states can be thought of as comprising behavioural, physiological, and subjective components (e.g., [42,44,45,46]). 

Although subjective components cannot be measured, affective states can be inferred by measuring the other components—behaviour and physiology—as ‘proxy indicators’. Some of these indicators of affect have not been validated specifically, but they are often observed in association with situations that at least appear to be rewarding or punishing. 

In light of the above, good animal welfare should be defined not only as the absence of negative emotional experiences, but also in terms of opportunities to experience positive emotions [39,46,47]. An animal is said to have ‘a life worth living’ when positive experiences outweigh negative experiences, and it is good practice to go beyond this and endeavour to facilitate a ‘good life’ for animals [48,49]. 

One approach to promoting a ‘good life’ is to provide laboratory animals with stimuli that meet their ‘species-specific’ needs, including environmental complexity and social stimuli for social animals [3,50,51]. This should enable them to engage in behaviours that they are highly motivated to perform, such as social exploration and bonding which, as indicated in our operational definition, are likely to induce positive affective states [43,46]. However, is it correct to think in terms of ‘species-specific’ needs with respect to sociality in male mice, or are their requirements in fact also gender-specific?

## 4. Benefits and Harms of Individual and Group Housing for Male Mice

This section aims to explore the current state of knowledge regarding sociality in male mice, drawing together literature that will help to better inform decision-making with respect to housing male mice, presented as benefits and harms of individual and group housing. 

### 4.1. Benefits of Individual Housing for Male Laboratory Mice

Clearly, individually housed male mice will never be attacked by another animal. In the literature, avoiding aggression is viewed by some experts as the *only* acceptable reason for individual housing (Vera Baumans quoted in [29]), for example if mice have been subjected to aggressive attacks, injuries or repeated social defeat. The most commonly given justification for singly housing male mice in our survey was because the individual animals had been aggressors or victims (Table 1 lists additional reasons). Removal from housing that had permitted these distressing experiences will clearly be beneficial.

Although this review addresses ‘male mice’ in general, it is also important to recognise that the likelihood of aggression can vary between strains. For example, Bisazza (1981) [52] found that social structure and behaviour, including aggression, in group-housed adult males differed greatly between strains. Swiss outbred males were highly intolerant of each other and established individual territories, whereas male BALB/c mice seemed more socially tolerant, formed groups that were organised into hierarchies, shared the same cage, and slept together in the same nest. In groups of C57BL/6 male mice, no fighting was observed and the mice appeared to live together without hierarchical organisation [52]. This between-strain divergence in social organisation may be the result of differences in behavioural ecology of the wild ancestors of laboratory mice; for example, males of the *Mus musculus domesticus* subspecies are more aggressive than those of the *Mus musculus musculus* subspecies [53]. Although most inbred strains used in the laboratory derive from the *musculus* subspecies, both genetic alteration and selective breeding for high (e.g., Turku Aggressive TA) and low (Turku Nonaggressive TNA; [54]) aggressiveness may have led to modifications in the social behaviour of different strains (see [55]). For instance, genotype-dependent differences in the level of social affiliation have been found in DBA and C57BL/6 mice, both *musculus* subspecies [56]. DBA mice were more likely to stay close to a familiar cage mate (within a 2.5 cm radius) in the middle area of an open field test, whereas C57BL/6 mice showed less affiliative behaviour [56]. Contrasting social behaviour (e.g., exploration, huddling, aggression) between males of the C57BL/6 strain and males of the BALB/c strain, the former spent significantly more time and also engaged more frequently in social interactions with an unknown stimulus mouse than the latter [57]. 

Of 144 respondents to our survey, there was an approximately 50:50 division between those who answered ‘yes’ and ‘no’ to the question, ‘In your experience, are the males of some strains too aggressive to group house?’ (73 and 71 respectively). An analysis of these responses according to the age at which mice are grouped suggests that this may be a factor, as responses agreeing that males of some strains are not too aggressive to group-house appear to be associated with grouping as littermates (shaded cells of Table 2). When asked which strains were too aggressive to group house, the most common responses were Balb/c (20 respondents), C57Bl/6 (17), ‘transgenic/GA’ (*n* = 11), FVB (8), SJL (7), and CD1 (3). Interestingly, C57Bl/6 and BALBc are often characterised as low/moderately aggressive [58,59,60].

Observations such as those made by Bisazza (1981) [52] suggest that male mice of strains with a high propensity to fight (e.g., FVB and Swiss/CD-1) may benefit most from individual housing [61,62], as repeatedly sustaining fight wounds will be painful and distressing. Although providing male mice of more aggressive strains with the opportunity to establish their own territories through individual housing may be compatible with natural behaviour in some respects, the absence of any other conspecifics at all, with no other signs of their presence such as urine marks of neighbouring males [21], is clearly not what males would experience in a natural territory (discussed in the next section). Whether, and to what extent, this is a welfare issue has not been evaluated to our knowledge. However, reviewing behavioural and physiological consequences of individually- and group-housing male mice, Brain (1975) [28] proposed that individual housing resulted in a low rather than high stress condition, perhaps because of the absence of challenge [28]. Hence, we can tentatively suggest that providing male mice with their own territory through individual housing, so they can effectively ‘secure’ alpha status, may be the right thing to do in aggressive strains that organise themselves in this way [62].

Individually housing male mice after they have been used for breeding seems to be more common practice, as expressed by some survey respondents. The experience of breeding promotes aggression, making it nearly impossible to regroup males post-mating without aggressive behaviour and associated consequences [21]. 

### 4.2. Harms of Individual Housing for Male Laboratory Mice

Individual housing has many effects on the behaviour and physiology of social animals [50,63,64]. In male mice, effects that have been noted include changes in behavioural, neuro-endocrinological, and neuro-physiological parameters (see [12]). More specifically, social separation from conspecifics (e.g., by placing animals in separate cage within the same room) has been shown to induce changes in corticosterone levels, the immuno-response [65], neurochemistry, drug metabolism, and reproduction (reviews in [28,61,66]). For example, when compared with male mice housed in pairs with ovariectomised females, individually housed males showed increased heart rate during periods of low and high motor activity and had more, but shorter, resting bouts, indicating disruption of the normal circadian sleep pattern [67].

Nevertheless, the extent to which these changes indicate poor welfare has been debated. Reviewing the effects of individual housing on mouse physiology and behaviour, Krohn et al. (2006) [66] argued that differences in these measures were of insufficient magnitude to have a significant impact on welfare. They suggested that any negative effects might be resolved, for example, through improvements in (non-social) enrichment. However, the conclusions of Krohn et al. (2006) [66] were hampered by the lack of standardised studies and variations in study design, test protocols, and housing conditions (e.g., stocking density, cage sizes and animal numbers), making it difficult to draw convincing conclusions about welfare implications [66]. 

More recent studies have investigated links between individual housing and measures designed to assess affective states that are anxiety- or depression-like. For example, socially deprived male mice exhibited increased anxiety and depressive-like behaviours in standard behavioural tests such as the open field test, elevated plus maze, forced swim test, and sucrose preference test, accompanied by higher levels of corticosterone and reduced brain BDNF levels (brain-derived neurotropic factor, a protein responsible for growth and survival of neurons; [68]). Mice in this study also showed increased frequency of self-grooming in the open field test, which has been suggested to reflect negative affect; indeed, self-grooming in rodents has been proposed as a relevant parameter for ‘modelling’ neuropsychiatric disorders in humans [69]. Although reports on self-directed behaviours in mice are sparse, the incidence of hair pulling (barbering) in laboratory mice, a type of abnormal repetitive behaviour, has been evaluated by Garner et al. (2004) [70] in a cross-sectional epidemiologic survey of a population of 2950 animals. The incidence of self-directed barbering was 5.7% for 88 singly housed mice and 0.6% in 1981 group-housed mice, suggesting that singly housed mice were more distressed. However, the incidence of partner-directed barbering in group housing was 7.5%, indicating that this behaviour is a problem in both single- and group-housing conditions, and female mice tended to perform this behaviour more often than male mice [70]. 

Further evidence that single-housing is deleterious to male mice lies in the fact that depriving male mice of any kind of social stimulation post-‘weaning’ (including auditory, olfactory and visual cues) is commonly used to generate mouse ‘models’ simulating neurological and psychological disorders in humans, e.g., depression and anxiety [71] or schizophrenia [72]. The consequences of the ‘social isolation syndrome’ in mice described by [73], comprising altered behavioural and neurochemical functions, clearly show that an inability to socially interact with conspecifics is likely to have a deleterious effect on the affective state of a social animal. 

On balance, individual housing is therefore not recommended as a standard protocol. However, where harmonious grouping is not possible, providing male mice with a more suitable environment through single-housing may be more favourable from the animals’ perspective. 

### 4.3. Benefits of Group Housing for Male Laboratory Mice

As mentioned previously, there is an argument that aggression is part of natural social behaviour, whereas living alone is not, and if this school of thought is followed, then male mice should generally be housed in groups [29]. The argument that the ‘freedom to perform natural behaviour’ is important for good welfare (e.g., [37]) is strengthened if evidence can be provided for its positive influences on physiological function and affective states. Indeed, interactions with conspecifics are essential for the welfare of social individuals, and this is facilitated by ensuring that the group is stable and of an appropriate composition [51]. For example, active engagement in social behaviours and activities by one animal can be a valuable source of novel stimulation, e.g., scent marking or digging can elicit exploration by other individuals [50,51].

Assessing the motivation for social contact can provide potentially powerful evidence for the welfare value of social housing to laboratory mice, because a stimulus that an animal is motivated to ‘work’ to obtain is rewarding, and is associated with positive affective states. Male mice do show motivation for social contact with other males; Van Loo et al. (2001) [74] reported that both subordinate and dominant BALB/cAnNCrlBR males, given a choice between a cage allowing visual and olfactory contact with their familiar cage mate or an empty cage, made their nests near their cage mate. This implied that they actively sought their proximity. This observation may be slightly surprising in view of the territorial nature of wild house mice and their tendency to exclude other males, and it remains unclear whether the dominants chose close proximity for company or control [74], nor is it understood why subordinates also opted for proximity. Group-housed males of this strain often inflict wounds to the tail and back of subordinates, but the male mice in this study had lived successfully together for a relatively long time, which may explain the results [74]. 

In the above study, the preference for company did not differ between littermates and non-littermates, but subordinates tested with an unknown conspecific of the same social status only showed a significant preference for the inhabited cage when lights were on, i.e., during the inactive period for these nocturnal animals. This suggested that familiarity—but not kinship—may be the main motivator for their social preference, so unfamiliar mice may sleep together but spend less time together when active [74]. 

Preference for social contact also appears to be linked to age [60]. The need for social contact during active periods seemed to increase in older male mice (BALB/c strain, 36 to 37 weeks of age) when compared with male mice of 6 to 7 weeks old. More time was spent exploring, and engaged in behaviours directed towards, a partition separating two familiar cage mates when the next-door cage was an inhabited cage than when it was empty [60]. 

However, when presented with the choice between social contact or nesting material, both young and old males exhibited a significant preference for nesting material over restricted contact with the familiar cage mate when they were engaging in sleep and sleep-related behaviours. This indicated a preference for thermal comfort and security during inactive periods. However, males strongly preferred each other’s company over individual housing and, when in full social contact, always nested together irrespective of social status [60]. 

Other research has shown that mice will work to gain access to a social partner, further indicating that they value companionship [75,76]. In these studies, mice released into a barren cage were able to access different resource cages, one of them containing a social partner, despite incurring increasing costs to attain these (e.g., lengthening transverses of shallow water, which is aversive to mice). However, is unclear whether visits to the social partner were motivated solely by the need for social interaction, or also by the need to patrol the area and access all available resources (e.g., food, shelter and space [75,76]). 

Likewise, male mice of different genetic backgrounds (C57BL/6J, DBA/2J, FVB/NJ, and B6129PF2/J hybrids strain) spent significantly more time in a chamber containing a stranger than in an empty chamber, and also expressed a preference for social novelty by choosing to spend more time with a stranger than with a known partner [77,78]. These responses may be strain-specific, as others have reported that socially housed male C58/J mice showed lower motivation to approach a stranger mouse than male C57BL/6J mice [79]. 

To ascertain whether mice showed motivation for social contact outside the contexts of competition, reproduction, parental care, or territoriality, Panksepp & Lahvis (2007) [80] utilised a social conditioned place preference (SCPP) task in which juvenile mice (A/J, C57BL/6J, DBA/2J, and BALB/cJ; 30–31 days of age) learned to associate environmental stimuli (two different types of litter) with either mixed-gender social housing or social isolation. Juvenile A/J, C57BL/6J, and DBA/2J mice approached and explored the stimulus signalling social contact to a greater degree than those associated with no social contact, indicating that social contact was desirable and the opportunity for social approach was perceived as rewarding [80]. However, juvenile mice from the BALB/cJ strain exhibited a significantly lower response to the above social conditioned place preference. These strain differences between BALB/cJ and C57BL/6J mice persisted when both were tested in a mixed-strain social group. This study is interesting in that, following the rationale of conditioned place preference studies, it suggests that most mouse strains associated environmental cues linked to the presence of conspecifics with a more positive affective state than cues linked to social isolation. 

In another conditioned place preference study, subordinate CD-1 males were found to prefer cues associated with the scent of their home cage and their dominant cage mate, compared with an empty cage with clean sawdust—but the dominant counterpart showed no such preference [81]. Although these findings suggest that subordinates find odour cues from their home cage including the scent of their dominant cage mate rewarding, it is questionable whether their preference reflects motivation for social contact or simply a preference for familiar odours compared to the unfamiliar odours in the cage with clean sawdust. 

In fact, a previous study by Gosling et al. (1996) [82] found that the response of subordinate male mice (TO strain) to scent marks varied in relation to their own competitive ability. Dominant male mice were usually attracted to scent-marked areas, while subordinates avoided them. However, subordinates who were physically larger than their dominant partners were more likely to choose a scent-marked substrate in a Y-maze choice against a blank substrate, whereas relatively small subordinates were more likely to avoid scent marks. These findings suggest that some male mice may seek company for competitive reasons (as also proposed by [60], which may explain the observations made by [81]. 

These studies suggest that mice prefer the ability to access social company over being housed alone, but there does not appear to be sufficient evidence to identify which motivator (e.g., thermal comfort, social novelty, or social contact) drives male mice to seek companionship. Moreover, it is unclear how these motivators interact with one another, or how they are modified according to life experience and genetic background of male mice. 

Nevertheless, the effects of socially housing male mice appear to be beneficial when measured in other ways, too. For example, Liu et al. (2013) [83] suggested that group-housing reduced levels of anxiety and depression induced by chronic restraint stress; singly housed mice showed increased immobility in the forced swimming test and spent less time in the elevated plus-maze test after stress treatment (e.g., repeated restraint) compared to group-housed mice. Earlier studies have reported that social interactions can positively influence health and stress responses indicating effects of ‘social buffering’ in socially living animals (a phenomenon in which conspecifics show a better recovery from distress when experiencing an aversive event together than when alone; [84,85]). 

Furthermore, in sibling mice, affiliative physical interactions were found to have an antinociceptive effect, by increasing the threshold to pain sensitivity, which was not observed between unfamiliar or unrelated mice [86]. Moreover, changes in nociceptive threshold were also found when siblings were reunited in adulthood after a long period of separation, but this did not occur when only olfactory cues of the siblings’ home cage were presented [86]. 

With regards to aggression, the benefits and harms of group housing are highly likely to depend on the social rank of an individual within the group. For example, the rewarding experience of victory during agonistic encounters could be considered as generating a positive affective state [87,88,89,90]. Indeed, positively reinforcing effects of aggression have been suggested, as male mice (OF1 strain) showed a conditioned place preference for an area where successful fighting had previously occurred [87] and expressed aggressive motivation by learning to self-initiate trials offering opportunities to attack [90]. Nevertheless, the consequences of receiving aggression and losing an agonistic encounter are of course likely to be aversive and punishing, generating a negative affective state (reviewed in [91,92]). The effects of inter-male aggression on physiological and behavioural parameters have been studied in some depth, as described in Section 4.4 below. 

### 4.4. Harms of Group Housing for Male Laboratory Mice

Whilst the social environment may have many beneficial effects on the well-being of social animals, it can also be a significant source of social stressors [93]. As mentioned previously, free-living mice tend to form despotic social systems where the presence of another male is not accepted by the dominant individual [28,62]. 

Inter-male aggression in the laboratory setting has been associated with the establishment of dominance relationships [62,94], with group size influencing both the level of aggression and the stability of the dominant–subordinate relationships [62]. For example, a high level of aggression was observed on initial grouping in small colonies of three to five males, but following this the dominant mouse effectively suppressed fighting among the subordinates. This resulted in a despotic hierarchy (i.e., a hierarchy with one dominant individual) in which aggression declined over the subsequent 21 days. However, in larger groups of nine and 12 males, there was ongoing aggression between subordinates and changes in dominant position occurred frequently [62]. 

Competition for dominance, and repeated trespassing into the social spaces of others, may therefore in general represent continual stressors and challenges for both the dominant male [62] and the subordinates, which will be considerably more frequent than under natural conditions. The inability of subordinates to escape when housed in laboratory conditions is an artificial situation that is not usually encountered in nature, although some ‘submissive’ males may be tolerated within territories in the wild; perhaps as a result of suppression of their marking behaviour [21,95,96]. Conversely, male mice of some strains may not show territorial behaviour in confinement and a lack of defendable terrain may decrease the level of aggression [11,19,97]. 

When male mice are group-housed and injurious aggression does occur, this is a serious welfare problem causing pain, distress, and in severe cases, even death. It is important to consider the causes of the aggression. In free-ranging male mice, aggression is a natural behaviour associated with the defence of territory and resources [98]. In the laboratory, however, food is available in abundance and direct competition for breeding opportunities in a group of males is clearly not a factor. The occurrence of fighting may be influenced by multifactorial components including the genetic propensity for aggression, the odour of females that encourages inter-male competitive behaviour [21], and procedures which disturb established group structures, e.g., introduction or removal of individuals when randomising [12,21]. 

Competitive aggression may further be explained by the disturbance of scent communication through husbandry practices. For example, when cages are cleaned, structures such as nests and latrine areas are destroyed, and the animals’ scent marks are removed. Cage cleaning has been identified as a cause of short term increases in aggression in male mice [10,59,99]. This is made worse by transferring litter from the used to the clean cage, as mice urinate on the litter and their urine contains hormones that can increase aggression; however, used nesting material contains hormones from glands in the body (such as the plantar glands in the foot pads) that inhibit aggression, so transferring nesting material from the used to clean cage has been shown to reduce aggression ([10]; but see below).

When aggression does occur, it is not only the losers that experience poor welfare. Male mice experiencing repeated positive fighting outcomes during daily agonistic interactions have been found to develop pronounced aggression, anxiety-like behaviour and impulsivity, disturbances in motivated and cognitive behaviours, and impairments of sociability [100,101,102]. They also displayed hyperactivity, attention-deficit behaviour, motor dysfunctions and repetitive stereotyped behaviours (e.g., jerks, rotations and head twitches), and pronounced self-grooming. Although it may be assumed that winning would be a positive outcome for the victor, these observations imply detrimental effects of repeated winning, combined with the associated stress of repeated fighting and the pressure of maintaining hierarchical status, collectively resulting in different types of psychopathy such as hyperactivity and depression, key symptoms of bipolar disorder [102]. 

#### Welfare Concerns beyond Aggression

Aggression clearly leads to serious welfare problems and is a critically important issue, but it is essential to recognise that there may still be negative welfare implications for subordinate males, even in the absence of obvious fighting or injuries. Various studies have reported behavioural and physiological differences associated with social defeat and subordination such as decrease in social interactions with unfamiliar conspecifics, aggression, and general activity [103,104], along with an increase in submissive and defensive behaviours (reviewed in [87], as well as physiological changes such as immune functions and metabolism [105,106]).

However, caution is needed in interpreting the effects of experimentally induced social stress, as these are not necessarily representative of long-term housing conditions. For example, studies based on the resident–intruder paradigm consisting of introducing a stranger (intruder) into the home cage of another (resident) are commonly used to induce social defeat, but are not representative of conditions in stable group housing and may be conducted in open field arenas, which induce stress and aggression.

In some home cage studies, links between social rank and indicators such as immune parameters and plasma hormone levels [107] may be less apparent in stable groups, indicating that living in a settled social group may not be stressful [105,108]. In contrast, studies on Swiss-Webster mice (classified as highly aggressive) found that dominants and subordinates in stable groups of ten male mice differed in anxiety-like behaviours as measured in the elevated plus-maze, with subordinates showing lower levels of anxiety than dominants. This may have reflected different facets of anxiety, if the dominants staying in more protective areas of the maze were displaying higher levels of risk assessment and avoiding possible danger, thus appearing more alert than the subordinates who expressed a contrasting coping strategy by exploring the open arms of the maze more frequently and for a longer period of time [109].

There are other physiological implications; for instance, subordination stress has been associated with decrease in general activity (e.g., exploration and locomotor activity, [100,104] and neuroendocrine changes linked with increased activity of the HPA (hypothalamic–pituitary–adrenal) axis [110]. In CD-1 male mice, social stress was found to have long lasting effects on spatial learning abilities in subordinates [111] as well as on responses to home cage odours in a place preference task, which could not be reversed by stopping social interactions and re-housing them singly [81]. Differences between these studies may be explained by strain differences in sociability (see benefits of individual housing); some males from strains with low levels of social acceptance may experience social housing as more stressful than others. Furthermore, the bladders of subordinate male mice in captivity have been reported to contain, on average, twenty times as much urine as those of the dominant males [95], which could lead to discomfort and health problems.

Taking all of the above into account, the animal welfare implications, and harms, of group housing thus appear to be highly variable and poorly understood at present.

### 4.5. Summary of the Benefits and Harms of Individual and Group Housing

The current literature suggests that it is, generally speaking, preferable from an animal welfare perspective to house male laboratory mice in groups. Housed with others, male mice are able to express a range of social interactions that are important natural behaviours. However, there are significant caveats associated with this statement. Living with a group of other males is not a natural situation for male mice, and is likely to cause significant stress to some individuals, and the best protocol in any given situation will depend upon a number of different factors. The authors suggest that group housing for male mice is the ‘less worse’ approach, but do not positively endorse this practice because male mice would naturally prefer to live with a group of females, not other males.

We emphasise the importance of regularly reviewing the literature and current practice for housing male mice, and ensuring that this is discussed within the facility, e.g., by the AWERB or Animal Care and Use Committee. Table 3 summarises key literature at the time of writing, to help facilitate such discussions.

## 5. Living Together Better 

It is clear from the current codes of practice, responses to our survey, and the economic implications of single vs. group housing given per diem costs of animal housing, that there is strong motivation to group house male mice and that facilities will continue to do so.

Of 147 survey respondents, 99 (67%) reported that it was general practice post-‘weaning’ to group house male mice, just 4 stated that these were routinely singly housed, and 44 (30%) stated that both housing conditions were applied. When asked whether they would like to find a way to group house male mice that were currently singly housed, 144 people responded and 118 (81%) said ‘yes’, as opposed to 26 who said ‘no’.

### 5.1. Physical Cage Design and Cage Cleaning

Housing protocols for male mice that aim to reduce fighting, achieve harmonious groups, and thus balance the behavioural and physiological needs of mice with scientific and economic demands, have been the focus of several studies (reviewed in [12]). Their findings, however, are somewhat contradictory because certain strategies have been found to ameliorate aggression in some studies but provoke fighting in others. For example, the transfer of soiled litter has been suggested in order to reduce aggression [118], but Van Loo et al. (2000) [10] stated that this is counterproductive and nesting material should be transferred (as mentioned above), whereas others recommend that cages should be completely cleaned and everything replaced [119]. Although the disturbance of scent cues through cage cleaning provokes aggressive behaviour, and agonistic interactions peak shortly after cleaning, complete removal of home cage odours does not disrupt established dominant-subordinate relationships whereas incomplete removal of odours can stimulate more aggression from dominant animals [21,119]. In terms of nesting material, the transfer of used material containing pheromones with aggression-modulating properties may be beneficial in groups where post-cleaning aggression occurs [10] and does not negatively influence animal behaviour in groups with low levels of aggression [59]. 

Increasing the environmental complexity of the home cage through enrichment is assumed to alleviate aggression, but effects vary with both strain and enrichment type, as some types of cage furniture seem to exacerbate intermale aggression [120]. For example, rigid shelters have been found to increase aggression as mice tend to monopolise these resources, whereas enrichment that can be manipulated (such as nesting material) was found to decrease aggression [10]. However, this appears to be strain-specific as others have reported opposite effects on NIH/S male mice, with nesting material enhancing fighting and shelters preventing it [121].

The availability and distribution of resources can also affect the activity and the aggressive defence of mice [98]. Focused defence of areas containing resources such as food, water, and nesting material has been observed in resident male mice in confrontation with an unfamiliar intruder, indicating that it is not the whole territory but areas containing valuable resources that are highly defended. That is, male mice housed in a cage with clustered environmental enrichments may show higher aggression when they have to compete for depleting resources, whereas dispersing resources may reduce aggression [122]. Forage feeding may reduce fighting as animals spent more time actively searching for food. However, foraging can also be regarded as competitive behaviour and providing group-housed male mice with the opportunity to forage for sunflower seeds had no effect on aggression [123].

Our survey asked which husbandry practices were employed to enable group housing of male mice, and the responses are set out in Table 4. Some other approaches were also entered as free text, of which the most common were the provision of chew sticks (12) and running wheels (4). 

### 5.2. Group Composition

Fighting has been observed less often in groups of three male mice than in groups of five or eight males [11]. As mentioned earlier, there is evidence that stable dominance hierarchies are established sooner in smaller groups [62]. The most frequently cited group sizes within the survey were ‘2 to 5’ (34 of 151 respondents) and ‘5’ (28 respondents). 

It is widely recommended to disturb group stability as little as possible (NRC 2011; Home Office 2014). Although brief periods of separation of individuals (6–12 h) due to husbandry or experimental procedures may not alter dominant–subordinate relationships [124] introducing or removing individuals in the longer term further elicits fighting. In rats, the removal of an individual has been found to lead to social stress among group members as evidenced by an increase in agonistic behaviours, audible vocalizations, and faecal corticosterone metabolite levels, indicating welfare impairments in the remaining animals [125]. Also, studies have suggested that familiarity is more important to successful regrouping than kinship, as non-littermates reared together from an early age show no difference in social interactions compared to littermates [74,126,127]. 

‘Weaning’ age and early life experience (e.g., repeated mixing of weaned mice before arrival at the laboratory) have been found to subsequently influence aggression in the home cage [123]. Male mice (C57Bl/6) removed from the dam at 14 days, seven days earlier than the typical ‘weaning’ age, were less likely to show aggression towards their cage mates than males removed at 21 or 28 days of age. However, others have reported that early ‘weaning’ can induce anxiety and aggression in adult mice, arguing that deprivation of mother-pup interaction from postnatal days 14–21 may significantly alter social behaviour in mice [113]. In the above study by Gaskill et al. (2017) [123], placing male mice into stable groups at ‘weaning’ had no effect on aggression levels in the mice as adults, and other enrichments believed to reduce fighting (e.g., scent treatment with lavender) had an unexpected negative effect as these increased aggression between male mice. The artificial smell possibly disrupted normal scent communication, causing an increase in aggression. 

Our survey asked at which life stage males were grouped. A total of 143 people responded, of which 114 reported grouping when male pups are separated from the dam (‘weaning’), 48 at pre-‘weaning’ as littermates, and 31 at post-‘weaning’ (more than one option could be selected). The most common age for grouping at, or after, ‘weaning’ was three to four weeks.

### 5.3. Other Husbandry Methods and Approaches 

A surprising finding of the study by Gaskill et al. (2017) [123] was that the method used to mark individuals for identification also appeared to have a significant impact on aggressive behaviour. Ear notched male mice were found to be more aggressive towards their cage mates than males marked with tail tattoos. The former is assumed to cause greater tissue damage which may result in a greater experience of pain that may, in turn, potentiate aggressive behaviour.

The findings of Gaskill et al. (2017) [123] show that spontaneous home cage aggression, despite stable grouping post-separation from the dam, can be triggered by a range of internal and external circumstances. For example, the above authors noticed behavioural variations in mice housed on different racks, with animals kept in cages on the rack side facing the active area of the experimental room showing more aggression. 

Interventions that are commonly regarded as low-stress, such as cage cleaning or visual checks, can also significantly increase intermale aggression (e.g., [10,59]), as can unpredictability of experimental procedures or routine husbandry practices [128]. Likewise, prolonged isolation and experimental procedures causing discomfort may lead to excessive aggression in male mice, which is why it is important to identify, and refine, all potentially uncomfortable, painful, or distressing life events, regardless of whether these are directly related to experimental procedures and their after-effects [59]. 

Some strains have been bred for their aggression, for example to study covariation of behavioural and physiological factors related to aggression (e.g., SAL short attack latency lines [129] or TA Turku Aggressive [54]), or have become highly aggressive as a side effect of inbreeding. Questioning the justification for using these strains and selecting docile strains for research or breeding purposes may therefore be options to reduce problems with aggression—provided that the characteristics of the alternative strain fits the purpose of the study, otherwise results will not be translatable and animals will be wasted. 

Cage dividers have been proposed, where male mice are housed in sensory contact but prevented from fighting [130]. However, vasectomised male mice (Hsd:NMRI; approx. 6 months of age) housed in sensory contact with another fertile male, but with a partition dividing the animals for ten days, showed clear indicators of distress such as increased heart rate, body temperature, and motor activity, and impaired nest building behaviour [131]. Indeed, established hierarchies do not cease when only physical contact is prohibited [96,104]. Desjardin et al. (1973) [95] noticed that urine pattern of dominant and subordinate males differed greatly under ultraviolet lighting. The visual evidence of dominant-subordinate relationships remained unchanged when males were kept in the same cage but separated by a grid [95]. In female mice, separation of pair-housed cage mates using a grid divider provoked a higher stress response during postoperative recovery (e.g., increased heart rate and behavioural alterations) compared to mice housed socially or in individual cages [132]. Consequently, lacking the opportunity to interact with others is likely to cause stress in female and male mice and is therefore not recommended.

Given the issues with housing male laboratory mice in groups, ways of providing alternative, compatible companions have been investigated. For example, housing intact males with ovariectomised females or castrated males has been proposed, although chasing and biting still occurred when castrated and intact males were initially paired [133]. Nevertheless, others suggest that castration could be acceptable, reasoning that the short-term pain and distress resulting from castration would be preferable to the long-term effects of aggression in group-housed male mice [134]. For highly aggressive strains such as CD-1, castration has been found to eliminate intermale fighting completely [135]. The above options would involve surgical procedures, creating obvious ethical and animal welfare issues (even if optimal surgical practice was to be followed) that would need careful consideration and a harm-benefit analysis. 

To conclude, the aggression-mitigating effects of any particular husbandry refinement may depend on strain type, other elements of the husbandry protocols and other external factors, which is why certain improvements may be practical in some cases but not others. It is important to be aware of this and ensure that any changes are carefully researched, monitored, and evaluated. 

## 6. Monitoring Animals and Welfare Assessment

Effective assessment of the welfare state of both singly and group-housed male mice, and prompt identification of any problems with aggression or distress, will help to optimise male mouse housing, husbandry, and care. To decide what is best from the animals’ point of view, relevant welfare indicators need to be defined, and these also need to be understood in context [5]. Table 5 suggests some ‘cage side’ behavioural indicators that may be helpful in monitoring male mice.

In addition to the signs stated in Table 5, any changes in behaviour may be significant; for example, in group-housed mice these could indicate a change in the group dynamic or time budgets, with welfare implications for subordinate animals in particular. Time budgets and synchronised activity pattern may be helpful indicators of social stress in social animals. However, there does not appear to be strong evidence of synchronised behaviour patterns in male mice, most likely because males would naturally avoid each other—although huddling and sleeping together might be observed [52,60], and could be good indicators of positive welfare. In wild populations, subordination behaviour has been associated with changes in activity within an individual’s time budget, as subordinates learn to avoid the dominant individual, becoming more active when the dominant animal is inactive [18]. Observations of animals occupying similar space, and using enrichment at the same time, may thus be useful as studies have shown that dominant males tend to limit the movements of subordinates by monopolising highly desirable areas (e.g., food, the nest site, or shelter; [98,122]. Behaviours like these may be easier to detect by animal technologists, who spend the most time with the animals and should have had the opportunity to learn about animal behaviour and how to monitor this. Aggression towards humans is also more likely to be noted by caregivers and may be a sign of negative welfare, although confounded by strain differences and handling techniques. For example, capture by the tail induces anxiety in mice, leading to negative interactions with the handler, whereas catching mice in cupped hands or a tunnel reduces anxiety and fear of the handler [138]. 

The use of nesting material and nestbuilding behaviour could also be practical welfare indicators, as suggested by Gaskill et al. (2013) [8]. Nest shape has been linked with aggression in group-housed male mice (C57BL/6), as cages with well-structured nests are associated with fewer wounds amongst the occupants, whereas poorly built or absent nests are associated with a higher wound rate. Nest building behaviour has been observed to be negatively affected after a painful surgical procedure without adequate analgesia [139,140], indicating that both physiological and psychological pain and distress can be indicated through this behaviour. Observation of the location of faeces might also be a practical assessment method, since defaecation within the nesting area is abnormal and has been associated with pain [139,141]. 

Aggression is not included in the table, because mild to moderate aggression is generally difficult to quantify, and there is no universal consensus as to what is an ‘acceptable’ level of fighting, so the appearance of wounds and injuries may not be a good indicator. Aggression can also peak temporarily after cage cleaning [10,119], but this may not represent normal conditions. It might be better to monitor how long fighting occurs, and how often, and set limits with respect to severity and duration. As aggression is often observed when groups are formed, setting a time limit after which fighting to determine hierarchy should have ended might be preferable. Poole and Morgan (1973) [62] reported that a dominant male emerges within the first 24 h and aggressive attacks from the dominant declined within 21 days after grouping. However, this may be unacceptable if the level of aggression is causing significant welfare problems in the interim. 

## 7. Conclusions

Humans have almost complete control over the availability, quality, and variety of environmental stimuli to which captive animals are exposed, including social partners, such that policies and practice with respect to animal housing, husbandry, and care can either compromise or enhance animal welfare [46]. This level of control is associated with a fundamental responsibility to minimise any restrictions on the extent to which an animal can satisfy their physiological and ethological needs, as reflected in UK and European legislation [6].

However, it is not possible to house male mice in a way that is compatible with their natural behaviour in the laboratory, and it is not possible to make sweeping statements regarding good practice for housing male mice. Whether group- or single-housing is better (or less worse) in any given situation is highly context-dependent according to a number of factors including strain, age, social position, life experiences, and housing and husbandry protocols. It is important to recognise this and research and evaluate what is preferable from animal welfare and ethical perspectives for a given strain and situation. The eventual protocol may also depend upon scientific requirements, but if these would compromise welfare these should be duly justified and given appropriate scrutiny by the AWERB/ethics/Animal Care and Use Committee and regulator.

Given the current state of knowledge, it is important to recognise the following principles:

- there is still much to learn about the behaviour of different mouse strains and how this is affected by housing, husbandry and care, life stage, and previous experiences;
- many ‘natural’ behaviours remain innate in lines of animals that have been bred in captivity for many generations, even though it may not be possible to express these in ‘standard’ laboratory housing (e.g., subordinates are unable to flee form aggressive attacks from the dominant mouse, migration out of the territory is impossible, complex mixed-sex social relationships cannot be established);
- the presence, or absence, of aggression is not the sole indicator of poor or good welfare in group housed animals—and preventing aggression does not automatically ensure good welfare; and
- Codes of Practice reflect current knowledge and good practice at the time of writing, but it may be necessary to review subsequent publications and come to an informed decision about alternative approaches to husbandry, in discussion with the regulator.

Moreover, understanding the social environment in which laboratory animals are kept is not only significant to their welfare but also directly affects experimental results and the quality of research [29,50]. From a scientific perspective, altered physiological and behavioural responses due to social deprivation, or social stress, may undermine the validity of research results and should be considered in study design [2,34,141]. 

The following action points (Table 6) should help to promote good welfare for male mice within establishments: 

### Future Research 

Given the very large numbers of male mice housed in laboratories worldwide, more research is urgently needed to better inform approaches to housing, husbandry, and care. In particular, studies that would advance understanding of the wider animal welfare impacts of single and group housing on male mice beyond aggression are essential; these should be conducted in realistic situations (as opposed to resident–intruder type paradigms) and aim to evaluate the influence of the factors set out in this paper, such as strain, life stage, age at grouping, housing and care, and an individuals’ position within the group (for group-housed animals). 

Given the variation between and within strains, it would be helpful for simple protocols to be developed to evaluate the behaviour and welfare implications of individual and group housing for specific strains, that could be used in-house (e.g., by animal technologists and researchers working together).

Improving animal welfare is clearly an essential consideration on legal and ethical grounds, but it is also important to evaluate the potential impact on the science of poor welfare due to single housing or social stress. This could have a significant impact on reproducibility and the potential benefit of (and thus justification for) individual studies.

Further research should also address questions about the motivation of male mice for seeking (and working for) the company of other males, distinguishing better between the thermal/social/information/novelty/competitive attractors for other males and assessing how these vary with life experience and genetic background. Moreover, the welfare state of male mice with different social status, and in different housing systems, should be evaluated using recently developed assessment tools such as cognitive bias testing or behavioural observations using continuous home cage monitoring [144]. 

Cognitive bias tasks can deliver valuable insights into animal emotions and their perception of situations [42], but most cognitive bias tasks used with rodents currently require intensive training and response behaviours may be sensitive to the test environment. However, some home cage cognitive bias tasks such as that suggested by Graulich et al. (2016) [145] are based on preferences for different substrates containing food rewards. Tests like these are applicable in the home cage and may hold the potential to assess affective states in mice without the confounds that can occur in an unfamiliar test area [145]. 

For practical and economic reasons, it may be advantageous to make use of already available data (e.g., data collected for other purposes in telemetry studies) to monitor activity and changes in time budgets. This may be especially useful to detect welfare implications when no obvious fighting occurs or at the onset of problems before the consequences become noticeable (e.g., changes in stress hormone levels, metabolic changes or the use of enrichment objects; [146]). In addition, post-mortem studies (e.g., organ weights) could be used to investigate physiological effects of social rank and housing. As pointed out in this review, comparison between studies can be hampered by differences in research protocols and husbandry procedures. More well-controlled and standardised research protocols are needed. 

Research suggests that lack of control over the environment has significant negative effects on stress and welfare [147]. It would be of interest to see whether studies could be set up in which male mice are able to choose their cage mates. Although allowing this level of choice is unlikely to be feasible in practice, such studies may give valuable insights into the behavioural and welfare needs of male mice, and help to better inform housing, husbandry, and care protocols that will go further to genuinely meeting the physiological and ethological requirements of these animals. 

## Figures and Tables

**Table 1:** Reasons given by survey respondents for singly housing male mice.

Justification for Single Housing	Number of Responses
Those individuals have been aggressors or victims	122
For scientific reasons—studies that require single housing	100
For procedure-related reasons (e.g., exteriorised devices)	71
Those strains are especially aggressive	38
This is routine housing for all male mice, to prevent aggression	9
That is how male mice prefer to be housed, according to their natural behaviour	3
Don’t know	3

Legend: more than one response could be selected; 358 answers were selected by 147 people.

**Table 2:** Perceptions that some strains are too aggressive to group housing against age at grouping.

Life Stage at Which Males Are Grouped	Yes, Males of Some Strains Are Too Aggressive to Group House	No, Males of Some Strains Are Not Too Aggressive to Group House
Pre-‘weaning’ as littermates	17	29
When they are separated from the dam (‘weaning’)	60	52
Post-‘weaning’	14	17

Legend: We use the term ‘weaning’ because this is widely understood, but in practice this refers to maternal separation as the mouse pups are permanently removed from the dam.

**Table 3:** Benefits and harms of individual and group housing for male laboratory mice.

	Individual Housing	Group Housing
Benefits	Own territory [28,112] Secured alpha status [62] No physiological or psychological distress resulting from social conflict (e.g., pain associated with injurious fighting) Safe environment for male mice in cases of high intermale aggression (e.g., breeding male mice)	Expression of natural social behaviours, including aggressive interactions [29] Ability to huddle for thermal regulation and body contact [60] Cognitive stimulation through social communication cues e.g., via scent marking [21,51] Social company as reward; evidenced by motivation to gain access to a social partner [60,74,75,76] Preference for social stimulation over social isolation [60,74,80,81,87] Strain-dependent sociability and social novelty [77,78] Rewarding effects of aggression (e.g., victory for the winners [87,88,89,90] Social buffering [65,113]; decrease in HPA activity and improved health through social support (review by [85])
Harms	Negative consequences of social deprivation (e.g., ‘social isolation syndrome’ apparent as changes in the brain, physiology and behaviour [64,73] Increased aggression towards unfamiliar conspecifics [114] Displacement behaviours and stereotypies to substitute social behaviours (e.g., hair barbering, [115]) Negative emotional effects (e.g., anxiety, depression, loneliness [114,116,117]	Social stress of dominance-subordination [18], leading to physiological and behavioural changes [28,92,106] Intermale aggression [9,12] Stress in the dominant male leading to behavioural aberrations (e.g., stereotypies, aggressive grooming (dominant mounts victim); self-grooming [101] Changes in activity with subordinates being active when dominant is inactive [18]; dominants restrict the movement of subordinates [62]
Conclusion	Individual housing offers the chance to fulfil some male-specific needs and avoid the risk of injurious aggression and social defeat, but at the expense of suffering from social deprivation	Group housing broadly provides males with opportunities to express natural needs as a social species and fulfil the desire to be with others, but there may be negative welfare implications depending on the position of an individual in the hierarchy

**Table 4:** Husbandry practices used for group housed male mice in a range of establishments.

Husbandry Protocol	Number of Respondents
Provide nesting material	140
Tunnels	119
Transfer nesting material from used cage to clean cage	114
Provide nest box **	59
Transfer litter (e.g., wood chip) from used cage to clean cage **	52
Forage feeding (part or all of usual diet)	50

Legend: 140 people responded and it was possible to select more than one option. There is evidence that the protocols denoted by ** actually exacerbate aggression in certain circumstances (see text).

**Table 5:** Welfare indicators for group- or individual-housed male mice.

Behaviour	Indicators of Good Welfare	Indicators of Poor Welfare
General activity	Mice follow circadian pattern; more active in dark period and less active in light period	Mice do not show expected activity pattern; may be less active overall, still for prolonged periods, or show no clear circadian rhythm
Cage space use	All animals use the cage space equally (G)	Some/all animals remain in very limited areas of the cage (e.g., in corners; wall hugging) (G) Animal is not using shelter after having used it before (S)
Feeding and drinking	Animal(s) feed and drink regularly and maintain healthy body weight	Animal is not feeding and/or drinking normally resulting in decrease/increase in body weight
Sleeping and resting	Mice huddle together whilst sleeping (G) Animal is resting in shelter in regular bouts (S)	Mouse does not rest with cage mates/shows a disturbed resting pattern (G) Animal is not resting in nest, unregular sleeping pattern (S)
Grooming	Normal self-grooming behaviour or allogrooming	Aggressive grooming of subordinates, hair barbering (G) Signs of alopecia, poor self-care (S)
Use of nesting material and nestbuilding behaviour	Well-built nest	Poorly constructed/abnormal nest or no nest
Enrichment use	Mice are all using enrichment items in roughly similar amounts across time and space (G)	Enrichment is monopolised by dominant animal/s, subordinate/s avoid enrichment (e.g., shelter) (G)
Other behaviours	Exploration behaviour, use of enrichment	Aggression, biting, stereotypies or abnormal repetitive behaviours (ARBs) (G) Stereotypies or other ARBs (S)
Cage appearance	Normal defecation and urination patterns	Unusual faecal/urine output (e.g., pooling of urine rather than marking, defaecation within nest site)
Response to human handling	Approaches caretaker when hand placed in cage	Animal(s) avoid/show increased aggression towards handler
Level of audible vocalisation	Low levels of audible squeaking (G)	Audible squeaking, often related to aggressive encounters (G)
Physiological measures		
Health indicators and body weight	Mice appear healthy, have normal body weight	‘Staring’ coat, raised guard hairs Wound and physical damage Low or high body weight Other signs of poor welfare (see references in legend)
Respiration rate	Normal (80–230 breaths per minute)	Too high/too low

Legend: Most of these indicators apply to both singly and group-housed male mice; those that do not are identified by S (single) or G (group). For further guidance on welfare assessment, see Hawkins et al. (2011) [136] and European Commission (2012) [137].

**Table 6:** Action Points for Animal Technologists, Researchers, Veterinarians, and AWERB/ACUC Members.

Find out more about natural mouse behaviour, e.g., by reading references and reviews such as Latham & Mason 2004 [16];Van Loo et al. (2003) [12]; Weber et al. (2017) [9].
Ask for a discussion and review of local practice for housing male mice as a topic for the AWERB (or AWB, ACUC if outside the UK). In the case of the AWERB, this is linked to several tasks including advising staff on accommodation and care, advising on the Three Rs, and providing a forum for discussion. This could include defining an ‘acceptable’ level and/or duration of aggression for group housed animals, and consideration as to whether male mice may have a ‘life worth living’ or a ‘good life’ at your facility.
Ask your local person responsible for ensuring that staff have access to species-specific information (the Named Information Officer in the UK) to research the behaviour of the strains of male mouse you currently use, and seek advice from internal and external colleagues on good practice for housing and caring for them.
If males are group-housed, review whether the housing protocols reflect current thinking regarding minimising the risk of aggression, e.g., with respect to group size, cage furniture, cleaning protocols, age at grouping, stability of groups, and quality and quantity of space.
Ensure that welfare assessment protocols for male mice, both day to day and during evaluations of housing systems, will capture both good and poor welfare.
If aggressive strains are routinely housed and/or used in the facility, question whether less aggressive strains could be used instead (e.g., as background strains in breeding programmes).
Check progress with the UK NC3Rs mouse aggression project and participate in similar initiatives. (nc3rs.org.uk/laboratory-mouse-aggression-study).
Ensure that any proposals for ‘solutions’ such as housing intact males with castrated males, or ovariohysterectomised females, are subject to full ethical review that gives due weighting to the harms and benefits for all the animals involved.
Further actions for researchers:
Discuss the housing protocol for male mice used in your studies with veterinarians, animal technologists, and care staff, and consult with people with expertise in mouse behaviour. Identify the animal welfare, ethical and scientific implications, and satisfy yourself that the chosen protocol is the optimal one.
If a study requires that animals are randomised, explore the potential to achieve this without disrupting groups (e.g., by identifying individuals, using minimally invasive techniques).
Report (and justify) the housing protocol in papers, posters and talks, according to good practice guidelines such as ARRIVE [142] or the Gold Standard [143].

## Supplementary Materials

The following are available online at www.mdpi.com/2076-2615/7/12/88/s1, File 1: RSPCA male mouse housing survey.
