# Supplementary material for: To Group or Not to Group? Good Practice for Housing Male Laboratory Mice
Source: Animals (Basel). 2017 Nov 24;7(12):88. doi: 10.3390/ani7120088 (PMC5742782; doi:10.3390/ani7120088)
Supplement: Supplementary file 1 [file animals-07-00088-s001.pdf]

RSPCA male mouse housing survey
Supplementary material to the review article: To group or not to group? Good practice for housing male laboratory mice

Summary: A survey was designed by Penny Hawkins, Sarah Kappel and Michael T. Mendl using Google forms, and was circulated throughout April 2017 via social media, colleagues in the field, and online discussion forums. It was aimed at a range of stakeholders including scientists, animal technologists, veterinarians, and members of Animal Welfare and Ethical Review Bodies (AWERBs), with the objective of ascertaining current practice, welfare issues, and views regarding housing male mice.

There were 14 questions; the first eight required an answer and the remainder did not require a response in order to submit the survey. Some were check boxes or multiple choice, whereas others enabled respondents to enter free text. It was possible to complete the survey anonymously, although most respondent chose not to and many were prepared to be contacted for further discussion. There were 147 responses to the first eight 'compulsory' questions, with a good level of response throughout. Most were from the UK, although some use terminology from elsewhere in the European Union, or the US. All responses have been reported, as this is a global issue. This document reproduces the survey questions, with the number of responses to each one and either a summary of all the responses to tick box/multiple choice questions, or examples of responses to the long answer questions.

The survey demonstrated that there was a fairly good level of awareness of natural mouse behaviour amongst respondents. The majority of people believed that male mice prefer to live with other mice and should be housed in groups; most respondents reported that mice are group-housed at their facility (although some housed mice both singly and in groups). Most were aware of the debate as to whether it is in the best welfare interests of male mice to house them with other males (94, as opposed to 51 who were not aware). Several would like more information on 'best practice'.

Most single-housing occurred because individuals had been aggressors or victims, or studies required this. There was a 50:50 split between respondents who did, or did not, find males of some strains too aggressive to group-house. Of those who did not, slightly more reported grouping males pre-'weaning' as littermates (as opposed to when they are separated from the dam ('weaning') or post-'weaning'). Most male mice are grouped at 'weaning'; if post-'weaning' then three or three to four weeks was the most common grouping age. The strains most cited as aggressive were BalbC and C57/Bl6. Most respondents wanted to find a way to group-house male mice who were singly housed.

The most cited group sizes were 'two to five' and 'five'. Common husbandry practices to enable group housing were providing nesting material, tunnels, and transferring nesting material from the used cage to the clean cage. Some commonly cited practices may be detrimental and cause aggression, e.g., providing a solid nest box and transferring litter from the used cage to the clean cage. 35 people reported aggression in group housed males; fighting or injuries were frequently reported, with many injuries consistently located at the same sites (genitals, base of tail, and back).

Equal numbers of people answered 'yes' and 'no' when asked whether their AWERB had ever discussed or reviewed the topic. Most AWERBs appeared to favour group housing. Many people felt that housing protocols would affect the science, if animals were stressed, but were unclear as to whether being the victim of aggression or being singly housed would be more stressful, or whether dominant or subordinate animals would be more stressed in a group situation. 45 reported changes in policy or practice with respect to singly or group housing male mice at their establishment, often following advice received from the Home Office Animals in Science Regulation Unit Inspector.

Survey responses

1. Role

This allowed people to tick multiple boxes, as many people identify with more than one role; hence $n = 256$ although 147 people responded.

Animal technologist	79
Named Animal Care and Welfare Officer (NACWO)	43
AWERB member	39
Scientist	29
Named Training and Competency Officer (NTCO)	17
Named Veterinary Surgeon (NVS) or deputy	16
Home Office Liaison Officer/Contact (HOLO/HOLC)	9
Named Information Officer (NIO)	7
Other management	7
Other veterinary role	5
Other	4
Establishment Licence Holder (ELH)	1
**Total**	**256**

2. Establishment type

University/medical school/hospital	82
Research institute (including public bodies, non-profit organisations, public health laboratories)	35
Pharmaceutical establishment	18
Breeding/supply establishment	4
Contract Research Organisation (CRO)	4
Other response	4
**Total**	**147**

3. Approximate number of mice housed in the facility at any one time.

1000 to 10,000	63
Over 10,000	43
100 to 1000	30
Up to 100	3
Don't know	8
**Total**	**147**

4. Do you believe that male mice naturally prefer to live with other mice?

Yes = 120, no = 20, don't know = 7, total = 147

5. Do you believe that male mice should, ideally, be group housed with other males in the laboratory, if breeding is not required?

Yes = 123, no = 12, don't know = 12, total = 147

6. Without looking it up (!) how do you think male mice (*Mus musculus*) normally live in the wild?

With a group of female mice and their offspring	72
With a group of other males and females, plus their offspring	58

With one female mouse and their offspring	6
Don't know	11
**Total**	**147**

7. Does your facility house male mice?

Yes = 147

8. If yes, is it general practice post 'weaning' to house these。

in groups	99
singly	4
both of the above	44
**Total**	**147**

Questions from this point onwards were optional.

9. For SINGLY housed male mice, what are the reasons for this at your establishment? Please tick all that apply: (This allowed people to tick multiple boxes, so n = 358.)

Those individuals have been aggressors or victims	122
For scientific reasons—studies that require single housing	100
For procedure-related reasons (e.g., exteriorised devices)	71
Those strains are especially aggressive	38
This is routine housing for all male mice, to prevent aggression	9
That is how male mice prefer to be housed, according to their natural behaviour	3
Don't know	3
**Total**	**358**

Some quotes:

One of our most common causes of singly housed males comes from the reduction of cage numbers (number of animals per cage) following genotyping.

Often males are single housed when used for timed mating (as studs). We would not usually take the risk of re-grouping the males after single housing. It would be good if there was a way to do this without being concerned for their welfare afterwards—it always seems a risk that they will fight, and should that risk be taken?

Any male who has ever bred should never be housed with another male.

9a. In your experience, are the males of some strains too aggressive to group house?

Yes = 73, no = 71, total = 144

An analysis of these responses according to the age at which mice are grouped suggests that this may be a factor, as responses agreeing that males of some strains are not too aggressive to group house appeared to be associated with grouping as littermates (shaded cells).

Life Stage at Which Males Are Grouped	Yes, Males of Some Strains Are Too Aggressive to Group House	No, Males of Some Strains Are Not Too Aggressive to Group House
Pre-'weaning' as littermates	17	29
When they are separated from the dam ('weaning')	60	52
Post 'weaning'	14	17

9b. if you answered 'yes', which strains? These answers were free text.

BalbC	20
C57 Bl/6	17
'Transgenic/TG/GA'	11
FVB	8
SJL	7
CD1	3
Axl	2
AKR	2
Nude	2
SCID	2
Swiss	2
CD4Tol	1
cd68	1
HHD Tg	1
Hu18	1
PD1	1
RAG	1
SAM	1
tg4510	1
tgHRAS	1
tg37	1
**Total**	**86**

9c. Would you like to find a way to group house those male mice that are currently singly housed?

Yes = 118, no = 26, total = 144

10. For GROUP housed male mice, what is the usual group size? NB Some respondents reported more than one group size.

2–5	34
5	28
3	12
3–5	12
4	12
10	8
3–4	6
4–5	6
2–3	4
6–8	4
2–4	3
2–8	3
9–11	3
10–15	2
'Variable'	2
2	1
2–9	1
3–6	1
5/6	1
2–26	1
No response	7
TOTAL	151

10a. What husbandry practices do you employ to enable group housing of male mice? Please tick all that apply: People could tick more than one, so *n* = 580.

Provide nesting material	140
Tunnels	119
Transfer nesting material from used cage to clean cage	114
Provide nest box **	59
Transfer litter (e.g., wood chip) from used cage to clean cage **	52
Forage feeding (part or all of the usual diet)	50
Responses below this are free text, in response to 'other'	
Chew sticks	12
Running wheels	4
'Environmental enrichment'	3
Mezzanine/shelf	3
Don't know	2
Transfer enrichment items from used to clean cages **	2
Tissue/paper towel on top of cage to pull through	2
House aggressive strains in trios	1
Cotton wool	1
Handle with clean gloves so previous scents aren't transferred	1
No toys in cage	1
Once separated, never regroup	1
Part clean system	1
Wean at the same time	1
Zoning' of cage to provide additional space	1

** There is evidence that these protocols actually exacerbate aggression in certain circumstances (see accompanying paper).

Quote: 'All breeders in the UK happily house males together with no problems'.

10b. At what life stage are the males grouped?

Options were (i) pre-'weaning' as littermates; (ii) when they are separated from the dam ('weaning'), (iii) post-'weaning'. Participants were able to select all options that applied at their facility.

When they are separated from the dam ('weaning')	76
Pre-'weaning' as littermates; and when they are separated from the dam ('weaning')	19
Pre-'weaning' as littermates	17
Post 'weaning'	11
Pre-'weaning' as littermates; when they are separated from the dam ('weaning'); and post 'weaning'	11
When they are separated from the dam ('weaning'); and post 'weaning'	8
Pre-'weaning' as littermates; and post 'weaning'	1
**Total**	**143**

Summary of totals for each option:

Pre-'weaning' as littermates	48
When they are separated from the dam ('weaning')	114
Post 'weaning'	31
**Total**	**193**

10c. if you ticked the second or third box (when they are separated from the dam; or post 'weaning')**, at how many weeks old?**

3 weeks, or 3 to 4 weeks	99
6–8 weeks, upon arrival, or by breeder	4
4 weeks	2
<3 weeks	1

Don’t know/depends	9
No response	28
Total	143

10d. Have there ever been any issues with male-male aggression, or behaviours of group housed males that suggest there could be a problem (e.g., abnormal repetitive behaviours, or stereotypies)?

Yes = 35, no = 29

This question prompted many responses, including quite detailed descriptions from those reporting aggression. Fighting or evidence of fighting (injuries) was frequently reported (in 30 responses), with many injuries consistently located at the same sites (genitals, base of tail, and back).

A few respondents mentioned that aggressive interactions appear to have arisen after a change in home cage sharing (e.g., reintroduction post-study or breeding). Stereotypic behaviours (circling, flipping etc.) were less frequently reported, but still appeared to be significant. There was also the perception amongst some that aggression was more common in particular strains (see questions 9a and b).

Selected comments:

- Lots of urine on cage furniture of mice likely to become aggressive in the future.
- Usually, behaviour issues manifest after fighting has occurred and mice have been separated, this is especially so with the SCID lines.
- When they are regrouped after being separated experimentally or if separated to breed.
- We house brothers together at weaning but these can get aggressive towards each other when sexual maturity is reached.
- Observed repetitive behaviours and increased fear of human interaction.
- We have male-male aggression frequently. We believe some is due to dominance, others we have yet to determine. I am currently trying to determine if there are undue stressors occurring prior to aggressive patterns being observed.
- Severe fights occur post-procedure, intense barbering, animal isolated from nest.
- See aggression when animals come out of IVCs into conventional cages, or there is a change in room/animals are put on study. Also have big issues with aggression when animals are bought in from commercial suppliers
- Aggressive behaviours noted on previous occasions when expanding group-housing was attempted.
- Aggression is common in a wide range of strains from 7 weeks of age onwards, often it is initially just one individual in the group which will be separated from the rest. Aggression is an instinctive response and can’t be modified in the confined environment of a laboratory cage. Social isolation of aggressive individuals is preferable to high levels of group stress and physical injury.
- The group seem to be agitated by the one running in circles, so when he is removed the aggression normally decreases
- Increased mortality due to fighting lesions (2 year studies)
- Aggression does occur in group housed males but with the exception of some strains, it is not usually predictable. I tend to think it is associated with clean out and the disruption of social cues.

11. Are you aware of any debate as to whether it is in the best welfare interests of male mice to house them with other males?

Yes = 94, no = 51, total = 145

11a. would you like to comment on this?

Sixty-two responded, with several people saying that they would like more information on best practice for housing male mice.

Selected comments:

I think that it is not ideal to house males together HOWEVER it is significantly better than single-housing them. The environmental stimulation another mouse provides greatly outweighs the negatives of housing two males together. While male mice do not live together in the wild and will fight upon contact, suggesting they should not be housed together in the lab, early pairing and the marked decreased in isolation stress make it worth it.

They are social animals, but compete with other males. In the wild, although I believe they live in mixed groups, it is skewed towards females. Many males die as a result of direct aggression from dominant males or indirectly by being ousted from the group (or deciding to leave) but then being predated or starving to death. It is difficult to know whether they would prefer to live with a threatening male or no one. On their own, they miss out on thermoregulation from huddling and the stimulation another mouse gives in a relatively barren life, and possibly the benefits of social buffering from stress. I suspect that when there is no aggression, they do prefer being with another male than no one; the difficulty arises when there is aggression.

It is difficult to balance the fighting risk and the risk of being lonely.

I expect that singly housing males for long periods of time (up to 2 years old) must have a negative impact on their well-being and should be avoided whenever possible

I think it is a current issue that needs extensive research so practices can be standardised and studies can be appropriately adapted.

I didn't know that it could be an issue to house unmated male litter mates together, assuming no aggression is evident.

In the wild, male mice live in groups but not with other (adult) males. Singly housed, they occasionally overgroom and show other behavioural abnormalities. On the other hand, male mice housed with their littermates occasionally fight. So I think there is no "ideal" solution. I try not to use bullies as breeders so as not to select for fight-promoting traits.

It concerns me that the evidence that wild mice rarely tolerate other males within their territory, the evidence (from Paul Brain's work in the 80's) that subordinate mice have significant physiological differences from dominant animals and the sheer welfare impact of the serious injuries inflected on a daily basis do not seem to be impacting the dogma that male mice should be housed together for their own "welfare".

There is scant evidence that single housed male mice suffer—the studies that exist only show differences (e.g., increased liver tumour incidence, but massively reduced skin tumour incidence due to lack of chronic fighting).

The evidence from post-surgical social housing is suggestive that there might be an issue, but unfortunately there is too little information on husbandry within those papers to determine whether this might be a lack of shelter causing isolation stress to be exacerbated or social housing providing the benefit of communal heat (mice seem to prefer to shelter in groups when there are no options to have an individual nest).

Very difficult to come to hard and fast conclusion we try and take each study as it comes and do the best for the mice. A lot depends on how 'disturbed' they will be (length of study, handling, dosing and so on) as I feel this doesn't help the aggressive attitude problem.

It has been suggested that male mice prefer solitary housing but I am not aware of any evidence to support this. The majority of sibling grouped males do not show extreme aggression but some strains/areas appear to be more prone to problems.

Single housing is expensive and aggression levels are not so high that I feel this extra cost is warranted. We don't keep any rodents in "natural" social structures or environments and we must question as to what effect inbreeding has on establishments of social hierarchy. I also thought that publications showed that although it may not be natural to house groups of males together they still preferred the social contact with a familiar male to being on their own.

Aggression is an instinctive behaviour and whilst its incidence can be reduced by selective breeding it cannot be eliminated. Male mice in nature establish a harem of several females and fight to exclude other males. In nature males without females live in isolation and have reduced survival chances.

I think there is insufficient data to fairly answer this question. I am not convinced that it is always less stressful to house male mice together than apart. I suspect numbers in the cage, the size of cage, caging conditions plus personalities of individuals (among a myriad of other things) will all make a difference.

I understand that mice are happier group housed. However older males can be very aggressive. As the trend to use aged mice continues it's difficult to call on a comparison with a wild mouse as the chances of them living to older than 2 years is slim.

If there is a way to reduce/stop the aggression and fighting between male animals when housed together then they could be housed. However currently we haven't found a way to reduce this and fight wounds interfere with the study results/parameters being measured therefore increasing variability of results, therefore more animals would be required on the study (or the study cannot be performed completely).

I agree that social housing for adult male mice can be beneficial, but only when certain conditions can be fulfilled. I believe that it is often not possible to fulfil these conditions in the context of experiments. I also feel that the debate on group housing has become over simplified by some, with a dogma developing that group housing is always best. In certain conditions, I think the cost of group housing in terms of aggression is not balanced by the benefit.

12. Has your Animal Welfare and Ethical Review Body (AWERB) ever discussed or reviewed this topic?

No = 67, yes = 66, total = 133

12a. would you like to comment on this?

Sixty-two also responded to this question, in free text. Of these, 30 said their AWERB (or Animal Care and Use Committee) had discussed, was reviewing, or would be considering group vs. single housing for male mice; 12 said their AWERB had not; and 28 did not know or did not give conclusive answers. Where outcomes were stated with respect to single or group housing, most AWERBs favoured group housing.

Selected comments:

Separation from aggression, breeding and attrition are veterinary reasons for single housing**.** If the vet sees it as the best interest for the animal and that is university practice, then the ethics board doesn't need to review the practice.

For mice, the AWERB has no directive to give regarding the social housing of mice other than it appears from evidence that male mice may benefit from single housing. Therefore, protocols requiring the single housing of mice do not require AWERB approval. It is recommended that if group housing is selected, this should occur in animals 23 days of age or younger. For the majority of studies, mice were gang-housed. Single housing was used (predominantly for males), where there was a requirement for older animals, and for strains where there is a known predisposition to fighting.

A committee was formed to investigate research done on singly housed mice. Some of those papers touched on male aggression. We were able to recommend nest transfer during cage change and have just begun a pilot.

It has been determined by our Institutional Animal Care and Use Committee (IACUC) that all animals (including mice) must have at least one cage mate unless the research protocol provides scientific evidence against pair/group housing. If a mouse is found to be aggressive towards cage mates, they may be separated to single housing, but must have the reason written on their cage card.

We are particularly concerned about aged males, who may be along for many months or years as once separated we cannot confidently re-introduce them to other males. We have attempted to look at longevity etc, but numbers are too small to be very meaningful and are often skewed by the animal's fate eventually being used in experiment rather than aged to its natural end of lifespan.

In the USA the GUIDE considers mice social so they are group housed unless scientific justification or aggression. As IACUC we struggle with should you just start all c57bl/6 males single to make science more repeatable or do you wait for trouble to occur.

Usually brought up by the NACWO or Animal Tech on committee or at mid-term reviews of project licence.

13. Do you have any views on the potential (positive or negative) effects on the science of either group housing (with other males) or singly housing male mice?

This question triggered 89 responses. Overall, there appeared to be a widespread perception that group housing would be preferable if it there was no risk of stress or 'unrest'. Most respondents believed that mice are a 'social species', and that single-housing should be avoided wherever possible, but awareness (from wherever it arose) and first-hand experience of male-male aggression led a number of respondents to suggest that group housing carries significant welfare risks, with several respondents wanting more scientific guidance on what would be optimal for the animals in their care. There was a general assumption that significant levels of stress in animals would affect the science, but it was uncertain as to whether single, or group housed animals would be more stressed—and what difference it would make if an animal was dominant or subordinate.

Selected comments:

I would think that a male mouse group housed without fear of continual fighting or unrest would be a more suitable scientific model than one housed in solitary confinement.

If fighting is not picked up straight away the animals could have increased levels of stress hormones (cortisol etc.) that may have an effect on the science. We have also found that animals that are singly housed tend to become obese quicker than group housed animals which may also have effects in procedures.

I think it would be negative to keep all males singly housed, it has been shown to increase heart rates and affect sleeping patterns and metabolism when singly housed which can have an effect on research.

For science, group housing may make for more normal models, and it also opens the possibility of sensitive repeated measures experimental designs, with cagemates acting as each other's controls. This can be a good use of space, maximising sample size. However, if treatment has to be confounded with cage (on treatment per cage), then single housing is possibly more efficient for the sample size, since cage has to be the experimental unit (so extra mice per cage add little to the statistical power).

I would like to know more about this; how groups live in the wild, effects caused different strain characteristics in laboratory mice, etc. Is there one answer fits all, or do decisions need to be made on a strain by strain basis or other criteria?

Single or group housing can affect studies involving brain development.

Some scientists are arguing that singly housing provides more reproducible results.

Aggression can lead to stress like symptoms and elevated blood results etc. the same as singly housed could also have adverse effects due to low interaction.

If males are fighting constantly, there will be an obvious rise in cortisol, and possible changes to both the systemic system and neurophysiology. I fear aggressive males may skew certain parameters in physiological data.

If results changed (e.g., upon group housing previously individual males), researchers would have to ask whether their existing data set was flawed, not necessarily blame the new conditions.

There is evidence from Paul Brain's work that subordinate male mice are significantly different physiologically from dominant animals—this adds a level of variability, as would the social stress if the group is not absolutely stable. Fighting causing separation of groups or losses of animals can effectively render a study invalid and either waste significant numbers of animals if the study is repeated, or result in poor science if the study continues.

I believe that singly housing mice increases stress levels which would affect scientific data.

Stress, but this could occur in both singly housed or group housed. Therefore it could distort study results—the need for consistency is very important.

I think this would depend a lot on the type of research and the specific research question. Single and group housing are known to affect different parameters in different ways so researchers should investigate these effects when designing studies.

Yes, some of our researchers are using behavioural measures as outcomes of dementia and Alzheimer's studies, and different responses may be seen between social and singly housed animals. I also wonder if there are less-obvious metabolic differences in groups, e.g., between a dominant and the subordinates, that could make their basal metabolism different from a sole male and introduce errors of repeatability etc.

It all depends on the experiment you are doing—for example group housing if recommended when performing behavioural experiments. However I had to single house some males as they were bullies—so not great for the single mouse but also for the mice that were attacked as their stress level went up. Now I preferentially use females for burrowing, males for fear conditioning

I think group vs. single housing is a potentially big issue. Mice can be stressed if isolated, yet some can be contented depending on how dominant or submissive they are. Mice can also become stressed or aggressive in group environments, yet some can be contented and settled in a group environment depending on how dominant or submissive they are. The chemicals released into the body caused by stress, and also the immune reactions caused by fighting wounds, can affect scientific results.

I believe the effects on science are probably neutral, except when aggression is present to the extent that it disrupts the experiment.

14. Have there been any recent changes in policy or practice with respect to group or singly housing male mice at your establishment, and if so what were the changes and what prompted these?

No = 45, no response = 53

Approximately two-thirds of participants either did not respond or responded negatively to this question. Of the remaining third, the responses were mixed, with several respondents mentioning that their facility adheres to the Home Office requirement of group housing wherever possible. Enrichment was a topic discussed by several respondents: singly housed mice requiring greater enrichment was mentioned on more than one occasion. While one respondent described tunnels being removed from cages as scientists suggested they may cause territorial behaviour, another described how they were running a study to see if the presence of tunnels affects plugging rate and therefore whether they could be used as enrichment for stud males.

Selected comments:

We ensure that at weaning all the male mice from the litter are housed in a single group. Once the genotypes are confirmed then the cage is reduced in size by the removal of those mice that are not needed, thus ensuring as they group we stay within the guidelines of the [Home Office] Code of Practice. We have also adopted a policy where we keep a companion animal if a male is going to be left on its own.

We tried only housing three to a cage in line with recent publications with little improvement seen. We use to take out the "bully" but with some lines if we see fighting we automatically single house the group as aggression tends to continue with other members taking the place of the aggressor.

We no longer regroup our male breeders after they have bred (sometimes grouped prior to breeding) or any mice once they have been separated do to aggression.

AAALAC (Association for Assessment and Accreditation of Laboratory Animal Care) mentioned the number of single housed rodents we have. As a result a committee was formed and now I have been named the enrichment coordinator to handle these issues. We are currently looking into more diverse nesting materials, minimizing single housing by getting larger rat cages and implementing a possible protocol review parameter that requires explanation for single housing along with plan.

The group sizes for males have been decreased and mixing policy has been adjusted after fighting issues during transfer

Males that are singly housed as studs have been given tunnels as enrichment to study if there is a comparison in plugging rates to those without tunnels. If there is no difference then we will provide tunnels as enrichment to singly housed stud males in the future. This was prompted by myself, as I questioned why they were not receiving tunnels as enrichment. I expected that stud males would be given more enrichment and not less, since they are singly housed. After I did not receive an adequate reason, we launched the study which will run for 3 months.

Working towards group housing males wherever possible, giving a male a non breeding female as a companion, added and different enrichment, Nod Skid mice have been re-paired at above 6 weeks old.

Changes were made for the CD1 strain as we were finding that males were fighting to the point of injury too often, which was affecting their welfare and the study they were in. CD1 males are now only group housed if the study requires it.

No changes that I am aware of. Caging conditions are fairly standard practice. Implant and stud/vas males are caged separately, but even when not presented with females or companion mice, will be aware of mice around them via their normal ultrasound communication. Individually caged mice in sound booths', or widely spaced from other mouse cages, would probably be the most isolated.

Not to clean out as much, to retain smells and prevent fighting. But some strains still fight, like the BALB/C.

We have recently had tried re introducing castrated males (which were originally litter mates) with advice from our NVS by mixing dirty bedding over a period of days. It was unsuccessful!

We've elected to work with males (CD1's) in a bid to reduce perceived wastage by the supplier. It's perceived that most users will prefer females, leaving many males being discarded. We feel we can do our part to take these animals and reduce (over)production.

We have been told that now we must inform the users of any singly housed males and ask if they can be used or culled to reduce the number we keep.

Group housing is pushed fanatically even when it potentially compromises a study.

We as an establishment removed tunnels from the caging, some scientists were concerned mice may become territorial and aggressive. We also noticed a reduction in aggression when we moved from isolators to IVCs.

All PPL (project licence) applications must now include justification for single-housing, whether for experimental or welfare purposes. These are therefore discussed by AWERB and increasingly attempts are being made to avoid single housing. This involves developing methods to try to increase the possibility of re-introducing males which have had to be separated temporarily (e.g., for a procedure or behavioural test).

We have been told that if we have to split animals for fighting that animals should be culled rather than split so that they are not housed on their own. I would rather not waste an animal so I would like to see evidence to show that it is detrimental to an animal to be housed on its own so that I feel like I am doing the right thing by culling the animal rather than wasting yet another life.

Choosing the vendor with the least frequency of aggression of the same strain. Study design and husbandry procedures that mitigates the frequency of aggression.

P.H., S.K. and M.T.M, October 2017
